# Edible Gels with Cranberry Extract: Evaluation of Anthocyanin Release Kinetics

**DOI:** 10.3390/gels9100796

**Published:** 2023-10-03

**Authors:** Rima Šedbarė, Valdimaras Janulis, Kristina Ramanauskiene

**Affiliations:** 1 Department of Pharmacognosy, Faculty of Pharmacy, Lithuanian University of Health Sciences, 50162 Kaunas, Lithuania; valdimaras.janulis@lsmu.lt; 2 Department of Clinical Pharmacy, Faculty of Pharmacy, Lithuanian University of Health Sciences, 50162 Kaunas, Lithuania; kristina.ramanauskiene@lsmu.lt

**Keywords:** *Vaccinium macrocarpon*, dissolution test, carboxymethyl cellulose, chitosan, carbomer

## Abstract

The bioactive compounds found in cranberry fruit are natural antioxidants, and their consumption reduces the risk of diabetes, cardiovascular disease, cancers, and urinary tract infections. Oral gels with cranberry fruit extract are a promising product that can ensure accurate dosage and release of the active compounds and are suitable for people with dysphagia. The aim of this study was to determine the effect of polymeric materials on the dissolution kinetics of cranberry fruit anthocyanins from gel formulations. Gel formulations were prepared using freeze-dried cranberry fruit extract with different gelling excipients: chitosan (G1–G3), sodium carboxymethylcellulose (G4–G6), and sodium carboxymethylcellulose combined with carbomers (G7–G9). The dissolution test showed that the release of anthocyanins from gel formulations G1–G6 and G9 was most intense within the first 10 min, with little change in the anthocyanin content of the acceptor medium afterwards. For the formulations based on carboxymethyl cellulose and carbomers G7 and G8, the amount of anthocyanins released into the acceptor medium gradually increased, which prolonged the release time of the active compounds. The test for the release of anthocyanins from the semi-solid systems through a hydrophilic membrane revealed that within the first hour, the total amount of anthocyanins released from the modeled gel formulations (G1–G9) was within the range of 6.02%–13.50%. The 1% chitosan (G1) gel formulation released the fastest and highest amount of anthocyanins (70% within 6 h). The other formulations showed a slower release of anthocyanins, and after 6 h, the amount of anthocyanins released from formulations G2–G9 was <57%.

## 1. Introduction

Cranberry fruits have a high nutritional value, and their raw plant material is promising due to its bioactive compounds, which can be applied in the development and production of natural colorants, preservatives, antioxidants, food supplements, and functional foods [1,2]. The inclusion of cranberry fruit in the diet enriches the body with natural antioxidants that have the effect of protecting the body’s systems against the development of chronic diseases and reducing the risk of diabetes, cardiovascular disease, cancers, and urinary tract infections [3,4]. A healthy and balanced diet is important for maintaining a person’s emotional and physical wellbeing, as well as for the prevention of the development of chronic diseases; thus, research into the potential of cranberry fruit for its use in dietary supplements and functional preparations is relevant and valuable.

One of the groups of bioactive compounds identified in cranberry fruit samples as having beneficial effects on human health is anthocyanins. Anthocyanins have been detected in fresh cranberry fruit from 25–65 mg/100 g [5]. About 90% of the total anthocyanin content in cranberry fruit samples consists of cyanidin and peonidin glycosides (cyanidin-3-arabinoside, cyanidin-3-galactoside, peonidin-3-arabinoside, and peonidin-3-galactoside) [6]. The anthocyanin fraction of cranberry fruit extracts has been shown to inhibit inflammatory processes [7], and cyanidin-3-galactoside isolated from cranberry extract has been found to be able to scavenge free radicals and to inhibit the oxidation of low-density lipoproteins [8].

When modeling pharmaceutical formulations, the solubility, stability, and bioavailability of anthocyanins are important factors to consider. At low gastric pH, hydrophilic anthocyanins are in the stable form of the flavylium cation, and are absorbed in the stomach via the carrier bilitranslocase [9]. Anthocyanin glycosides are rapidly absorbed, but their determined amount in blood plasma is low and only constitutes 1% of the anthocyanin intake [10,11,12]. Although the bioavailability of anthocyanins is low, their plasma concentrations are sufficient to induce changes in signal transduction and gene expression in vivo [10]. Anthocyanins that are not absorbed in the gut are transformed into less stable forms (including carbinol bases, chalcones, quinonoid bases, and anionic quinonoid bases) due to changes in pH, and are metabolized, especially by the gut microbiota, to produce smaller molecules, such as protocatechuic acid, vanillic acid, or ferulic acid [13]. This often leads to an increase in the amount of the strains of the commensal gut bacteria *Bifidobacterium* spp., *Lactobacillus* spp., and *Actinobacteria* [14]. Commensal intestinal bacteria are significant for enhancing the metabolism of flavonoids, improving intestinal barrier function, enlarging mucus secretion, producing short-chain fatty acids, and regulating lipid metabolism [15].

The low bioavailability and stability of anthocyanins limit their effect, and thus, when modeling pharmaceutical formulations, it is important to achieve a targeted local delivery of the active ingredient with good bioavailability and a controlled release rate [16]. The growing interest in the development of hydrogels, due to the ease and affordability of their production, is encouraging researchers to look for their application in oral pharmaceutical forms. Most medicines and food supplements are taken orally. Oral gels, which are suitable carriers for the incorporation of the active substance, are currently gaining increasing acceptance [17]. It should be noted that the incorporation of the active substances in a semi-solid matrix avoids swallowing problems, which is particularly important in pediatrics and geriatrics [18]. Precise dosing of oral gels in single-dose containers offers advantages over their standard liquid pharmaceutical forms. One of the important requirements for oral dosage forms is the dissolution kinetics of the active substances from the dosage form. When modeling solid oral dosage forms, it is important to ensure the kinetics of the active substance, as only dissolved substances can pass through the body’s biological membranes and enter the systemic circulation to produce the desired pharmacological effect [19]. It should be noted that dissolution test methodologies for some oral pharmaceutical forms, such as capsules, tablets, or suspension, have been well developed, and their application is mandatory for the quality assessment of other oral dosage forms [20]. Given the current proliferation of new oral dosage forms, researchers face new challenges in assessing their quality. The scientific literature contains research data on gels that were administered orally [21]. The release of the active substance from the semi-solid gel matrix depends on the choice of gelling agent, its concentration, and the viscosity of the formulated gel. An in vitro release study using Franz diffusion cells was used to assess the kinetics of the release of active compounds from a semi-solid matrix [22]. A synthetic membrane is often used as an inert support membrane [20]. This method allows for assessing how much of the active substance is released from the simulated substrate and how much of it passes through the semi-permeable membrane into the selected acceptor medium. In view of the fact that gels may be administered orally, the application of the dissolution kinetics test may provide additional information on the kinetics of the release of the active compounds from the gel matrix under digestive tract conditions simulated in vitro. One of the important objectives of the present study was to evaluate the influence of different gelling agents on the release of the active compounds of cranberry extracts from semi-solid gel matrices by means of in vitro dissolution and release tests.

It is important to select the appropriate polymers and their amounts to ensure the stability and release of the active substance from the semi-solid matrix [23]. Both natural and synthetic polymers are used in the manufacture of hydrogels [23]. In this study, hydrophilic gels with suitable organoleptic properties and potential for oral administration were selected. The polymers selected for the production of gels in this study were chitosan, carbomer, and sodium carboxymethyl cellulose. Chitosan is a natural cationic copolymer that is suitable for the formation of hydrogel structures [24]. This polymer is hydrophilic in nature and can be degraded by human enzymes, leading to its biocompatibility and biodegradability [24]. Recently, a growing body of research data has been published on the application of carboxymethyl cellulose in oral delivery systems [25]. Carboxymethyl cellulose is widely used in oral drug delivery systems due to its properties, such as its hydrophilicity, adhesiveness, non-toxicity, and the ability to form gels [25]. Carbomer is one of the most commonly used polymers in hydrophilic gels and liquid dosage forms as a modifier of rheological properties [18]. Carbomers readily absorb water on contact, become hydrated, and swell [26]. In tablets, carbomers are used as binders and control the release of the active drug [27]. In addition, bioadhesive oral formulations containing carbomers increase the gastrointestinal transit time of medicines and improve their bioavailability [28].

It is relevant to study the impact of polymeric materials on the dissolution kinetics of the phenolic compounds of cranberries. For this reason, the purpose of the present study was to formulate gels containing cranberry extract with anthocyanin complex and to carry out a biopharmaceutical evaluation of the gel formulations.

## 2. Results

In this study, we investigated the composition of anthocyanins in the dry extract of large cranberries. The results of the qualitative and quantitative composition of anthocyanins and anthocyanidins are presented in Table 1. In total, eleven anthocyanins and anthocyanidins were identified in the cranberry extract: delphinidin-3-galactoside, cyanidin-3-galactoside, cyanidin-3-glucoside, cyanidin-3-arabinoside, peonidin-3-galactoside, peonidin-3-glucoside, peonidin-3-arabinoside, malvidin-3-arabinoside, malvidin-3-galactoside, peonidin, and cyanidin. In the extract of large cranberries, the predominant anthocyanins that comprised 96.57% of their total content were cyanidin-3-galactoside (1469.46 ± 22.0 µg/g), cyanidin-3-arabinoside (947.66 ± 14.2 µg/g), peonidin-3-galactoside (1976.20 ± 29.6 µg/g), and peonidin-3-arabinoside (668.11 ± 10.0 µg/g). Other anthocyanins that comprised 3.43% included the following: delphinidin-3-galactoside (10.75 ± 0.2 µg/g), cyanidin-3-glucoside (32.79 ± 0.5 µg/g), peonidin-3-glucoside (105.35 ± 1.6 µg/g), malvidin-3-arabinoside (9.73 ± 0.2 µg/g), malvidin-3-galactoside (7.73 ± 0.1 µg/g), peonidin (5.17 ± 0.1 µg/g), and cyanidin (8.37 ± 0.1 µg/g).

Nine different gel formulations (G1–G9) were produced using freeze-dried dry cranberry fruit extract. Formulations G1–G3 used chitosan as a gelling agent, formulations G4–G6 used sodium carboxymethyl cellulose, and formulations G7–G9 used sodium carboxymethyl cellulose in combination with a carbomer as a gelling agent. The test produced red homogeneous gels with a faint cranberry odor (Table 2). The pH of the gel formulations produced was acidic, with pH values of < 3 in the G1–G6 gel formulations and higher pH values (>3) in the carbomer gel formulations.

**Figure 1:**
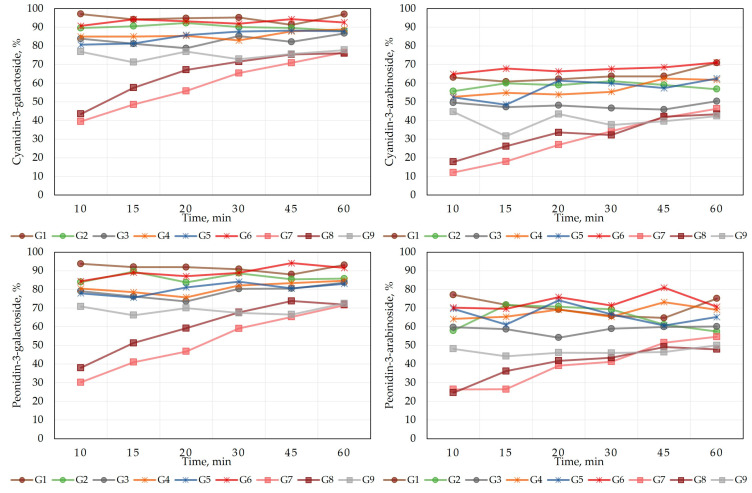
Dissolution kinetics of individual cranberry anthocyanins in acceptor media from gel formulations. Values are presented as means (n = 3).

**Figure 2:**
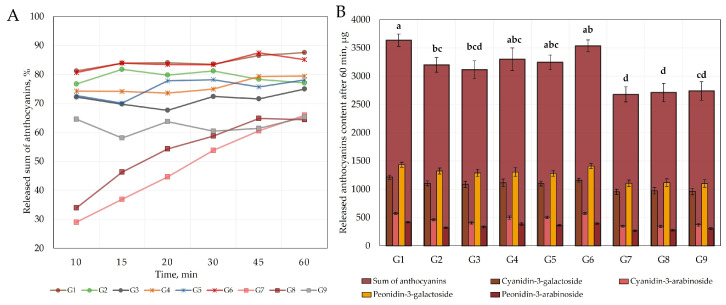
(**A**) Dissolution kinetics of cranberry anthocyanins in acceptor media from gel formulations. (**B**) Released anthocyanin content after 60 min from gel formulations. Different letters indicate significant differences (*p* < 0.05) between sum amounts of anthocyanins in the tested gel formulations, and gel formulations with same letter were not significantly different (*p* > 0.05).

To assess the quality of the oral gel formulations, the kinetics of the dissolution of anthocyanins was determined. To establish the dissolution kinetics of the modeled gels, a dissolution test was performed using a dissolution paddle device, whose mechanism of action mimicked the physiological conditions of the organism in vitro. The results of the dissolution kinetics are presented in Figure 1 and Figure 2. During the dissolution test, we evaluated the dissolution kinetics of the predominant anthocyanins (cyanidin-3-galactoside, cyanidin-3-arabinoside, peonidin-3-galactoside, and peonidin-3-arabinoside) in the cranberry fruit extract from the gelled matrix in the acceptor medium. The other anthocyanins and anthocyanidins detected in the extract were not able to be determined, as their concentrations in the acceptor medium were below the limit of detection (LOD) or limit of quantification (LOQ).

The kinetics graphs of the dissolution test (Figure 1) visualize the amount of individual anthocyanins released in the acceptor media from the modeled gel formulations (G1–G9) at different time points. During the dissolution test, the dissolution kinetics of the anthocyanins peonidin-3-galactoside, cyanidin-3-galactoside, cyanidin-3-arabinoside, and peonidin-3-arabinoside followed a similar pattern, but it is noteworthy that the amount of anthocyanins arabinosides released from the gel formulations was from 16% to 36% lower than the amount of galactosides (Figure 2). After 60 min of dissolution, the gel formulations G1–G9 released from 76.0% to 96.9% of cyanidin-3-galactoside, from 71.6% to 93.1% of peonidin-3-galactoside, from 42.3% to 71.1% of cyanidin-3-arabinoside, and from 47.9% to 75.1% of peonidin-3-arabinoside.

The kinetics graph of the dissolution test (Figure 2A) visualizes the total amount of anthocyanins released in the acceptor media from the modeled gel formulations (G1–G9) at different time points. The release of anthocyanins from gel formulations G1–G6 and G9 was most intense at the 10 min time point, with little change thereafter. The accuracy of sampling using a dissolution tester depends on the mixing speed of the medium, the sampling location (distance from the container wall), the distribution of the sample in the medium, the dissolution kinetics of the compounds, interaction with excipients in the acceptor medium, and other factors. The comparison of the anthocyanin contents of the G1–G6 and G9 formulations in the acceptor medium at baseline and after 60 min showed no statistically significant difference (*p* > 0.05). The fluctuations in the curves of these formulations presented in the graph do not have statistically significant differences and can be considered deviations of this study. During the dissolution test, the amount of anthocyanins released from the gel formulations based on carboxymethyl cellulose and carbomer G7 and G8 into the acceptor medium gradually increased, which prolonged the release time of the active compounds. The highest total amounts of anthocyanins, 85.1% and 87.5%, were released from the chitosan gel formulation G1 and the sodium carboxymethyl cellulose gel formulation G6, respectively. The carboxymethyl cellulose and carbomer gel formulations G7, G8, and G9 released statistically significantly lower amounts of anthocyanins into the acceptor medium (65.9%, 64.4%, and 65.2%, respectively).

The anthocyanins content released into the acceptor medium from the 5 g gel formulations (G1–G9) varied from 2675.7 ± 133.8 µg/g to 3637.5 ± 109.1 µg/g (Figure 2B). Gels G1, G4, G5, and G6 released the highest total amount of anthocyanins and were not statistically significantly different from each other after 60 min (*p* > 0.05). The sum of anthocyanins released after 60 min from gel formulations G7–G9 was statistically significantly lower than that released from gel formulations G1, G4, G5, and G6. The extended-release gels G7 and G8 released by up to 24.6% and 28.6% less anthocyanins than the other gels in the dissolution test, respectively.

**Figure 3:**
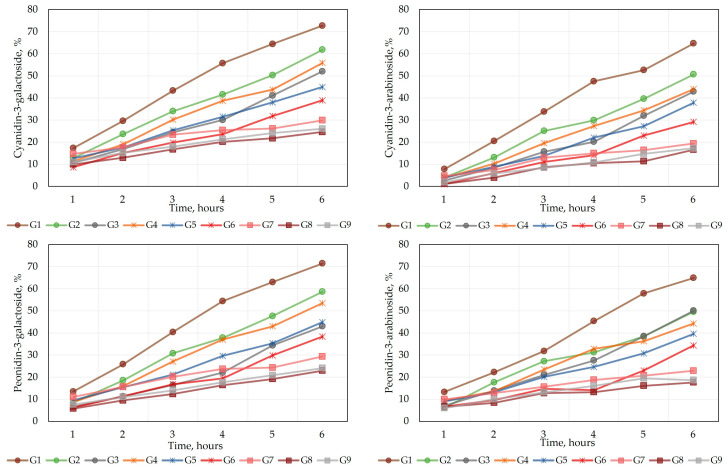
Cumulative release profiles of individual cranberry anthocyanins across a cellulose membrane. Values are presented as means (n = 3).

**Figure 4:**
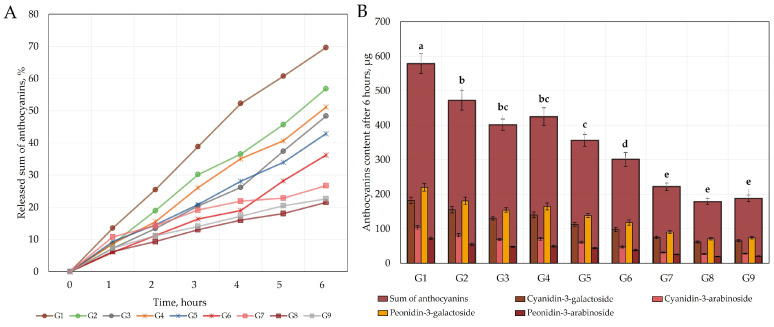
(**A**) Cumulative release profiles of cranberry anthocyanins across a membrane. (**B**) Released anthocyanins content after 6 h across a membrane. Different letters indicate significant differences (*p* < 0.05) between sum amounts of anthocyanins in the tested gel formulations, and gel formulations with same letter were not significantly different (*p* > 0.05).

To assess the release kinetics of the oral gel formulations, a release test was carried out using Franz diffusion chambers with natural cellulose dialysis membranes. The release of anthocyanins from the gel formulations allows for the evaluation of the passage of anthocyanins through the hydrophilic membrane barrier under in vitro conditions. The results of the kinetics of the transition from the diffusion cell across the cellulose membrane to the acceptor medium are shown in Figure 3 and Figure 4.

The kinetic graphs of the membrane permeation assay (Figure 3) visualize the amount of individual anthocyanins that entered the acceptor medium across the hydrophilic membrane from the modeled gel formulations (G1–G9) at different time points. The release of the anthocyanins peonidin-3-galactoside, cyanidin-3-galactoside, cyanidin-3-arabinoside, and peonidin-3-arabinoside across the membrane was carried out over a period of 6 h. After 1 h, ranges varying from 8.7% to 17.3% of cyanidin-3-galactoside, from 1.0% to 7.8% of cyanidin-3-arabinoside, from 5.8% to 13.5% of peonidin-3-galactoside, and from 6.0% to 13.3% of peonidin-3-arabinoside were released across the membrane from the G1–G9 formulations into the acceptor medium. Within 6 h, ranges from 24.6% to 72.7% of cyanidin-3-galactoside, from 16.6% to 64.7% of cyanidin-3-arabinoside, from 23.0% to 71.5% of peonidin-3-galactoside, and from 17.6% to 64.9% of peonidin-3-arabinoside were released from the G1–G9 formulations into the acceptor medium across the membrane.

The graph of the kinetics of the membrane permeation test (Figure 4A) visualizes the total amount of anthocyanins that entered the acceptor medium through the hydrophilic membrane from the modeled gel formulations G1–G9 at different time points. This study revealed that the maximum release of total anthocyanins from gel formulations G1–G9 was observed after 6 h. The total amount of anthocyanins released from the edible gel formulations G1–G9 across the plasma membrane after 6 h varied from 178.75 ± 8.9 µg/g to 578.83 ± 28.9 µg/g (Figure 4B). The highest total amount of anthocyanins (578.83 ± 28.9 µg/g) was released from the gel formulation G1, which accounted for 69.64% of the total anthocyanin content in the gel (*p* < 0.05) (Figure 4). The lowest total amount of anthocyanins (178.75 ± 8.9 µg/g) was released from the gel formulation G8, which accounted for 21.51% of the total anthocyanin content in the gel (Figure 4). The total anthocyanin contents of the formulations prepared using the gelling agents sodium carboxymethyl cellulose and carbomer (G7, G8, and G9) did not statistically significantly differ between the formulations and were found to be the lowest (26.70%, 21.51%, and 22.62%, respectively, *p* > 0.05).

This study showed that the selected polymers changed the kinetics of the release of anthocyanins from gel formulations with cranberry extract. Gels G7–G9, containing the excipients carboxymethyl cellulose and carbomer, showed a statistically significant longer dissolution rate and time of release into the acceptor medium.

## 3. Discussion

Hydrogels are polymeric structural systems with a high degree of swelling, which allows them to retain a large volume of liquid and a soft consistence [29]. The biocompatibility and degradability of hydrogels, the possibility to modify their mechanical properties to incorporate various active substances into gel formulations, and the ability to simulate the controlled release of substances from gel systems increase the potential of such systems and their application in the development of new formulations and products [29]. Such products are particularly important for people who have swallowing problems and cannot take tablets or capsules [18].

The development of pharmaceutical formulations containing anthocyanins is challenging due to their instability and low bioavailability [16]. The interaction between anthocyanins and gel-forming excipients is important for stabilizing and protecting anthocyanins from degradation in the biological environment and for modifying their release from gel formulations [30]. This study investigated the influence of the gelling agents chitosan, sodium carboxymethylcellulose, and carbomer on the solubility and bioavailability of anthocyanin-containing gel systems.

Chitosan is a linear polysaccharide obtained via the deacetylation of chitin [31]. Chitosan is a polycationic biopolymer that has good biocompatibility and biodegradability, is non-toxic, and has antibacterial properties [32]. Chitosan forms gel structures at pH levels below 6.2 [33], and thus cranberry extract with an acidic pH is suitable for the production of chitosan-containing gel formulations. Anthocyanins in cranberries form a flavylium cation in acidic media, which interacts with the polymeric structures of chitosan and enters the gel structures formed by chitosan [16]. Interaction between the hydroxyl groups of anthocyanins and the amine groups of chitosan via hydrogen bonding stabilizes the gel system but may also lead to electrostatic repulsive interactions between the positively charged flavylium cation of anthocyanins and the positively charged chitosan polymer [33,34,35]. The chitosan gel structure dissolves more readily and rapidly in aqueous acceptor media due to the formation of hydrophilic bonds between the positively charged amine groups of the chitosan and the water molecules, which results in a more rapid release of the active ingredient from the chitosan-containing gel system [33]. This explains why the chitosan-containing gel formulations (G1–G3) showed a rapid release of anthocyanins, with the highest amount of anthocyanins being released within 10 min (Figure 2). Increasing the chitosan content in the gel formulations (1% chitosan gel (G1) < 2% chitosan gel (G2) < 3% chitosan gel (G3)) increased the amount of chitosan polymeric structures that interacted with the anthocyanins, which may have led to a decrease in the amount of anthocyanins released at 60 min when comparing the G1 gel formulation to the G2 and G3 gel formulations (Figure 2).

The polysaccharide sodium carboxymethyl cellulose is a cellulose derivative which is highly soluble in water and has stabilizing and viscosity-modifying properties [36]. Sodium carboxymethyl cellulose is an anionic polysaccharide that can attract positively charged anthocyanins and stabilize them in the polymer structure [37]. In this study, we tested gel formulations with 2% sodium carboxymethylcellulose (G4), 3% sodium carboxymethyl cellulose (G5), and 4% sodium carboxymethyl cellulose (G6). The results showed that anthocyanins were released rapidly (within < 10 min) from these gel formulations, which was deemed to be due to the good aqueous solubility of sodium carboxymethyl cellulose and anthocyanins, and to the fact that the neutral aqueous medium changes the charge of the anthocyanins and decreases the interaction between the anthocyanins and sodium carboxymethyl cellulose [25,38,39]. The concentrations of sodium carboxymethyl cellulose in the gels studied did not have any statistically significant effect on the total amount of anthocyanins released from the formulations (*p* > 0.05). The amount of anthocyanins released by the sodium carboxymethyl cellulose gel formulations (G4–G6) after 60 min was not statistically significantly different from the amount of anthocyanins released by the chitosan gel formulations (G1–G3) after 60 min (*p* < 0.05), and the kinetics of their dissolution was similar (Figure 2).

The gel formulations with carbomer and sodium carboxymethyl cellulose showed slower dissolution rates and statistically significantly lower anthocyanin release rates (Figure 2). Carbomer is a polyacrylic acid polymer with a high molecular weight [27]. Carbomer is in the form of a solution under an acidic pH and forms a low viscosity gel at alkaline pH levels [27]. In this study, cranberry fruit extract was dissolved within the range of 0.5%–1.0% of the formulated carbomer gel to simulate gel formulations, but such gels were liquefied by the acidic pH and are not suitable for modeling as oral gels. The combination of carbomer and sodium carboxymethyl cellulose in the gel formulations allowed for the modification of the dissolution kinetics of anthocyanins. In the gel formulations, the rate of dissolution kinetics of anthocyanins increased with increasing sodium carboxymethyl cellulose concentration: 1% carbomer with 2% sodium carboxymethylcellulose (G7) < 1% carbomer with 3% sodium carboxymethyl cellulose (G8) < 1% carbomer with 4% sodium carboxymethyl cellulose (G9). While the dissolution of anthocyanins was prolonged in gel formulations G7 and G8, the total anthocyanin content released from formulations G7–G9 after 60 min was not statistically significantly different from each other, and amounted to about 65% of the total anthocyanin content (*p* > 0.05). The prolonged release kinetics of anthocyanins in the compounded gel formulations was due to the high molecular weight of the carbomer and high cross-linking, which increases the viscosity of the gel and modifies the rate of release of the active compound [40,41].

Improving the bioavailability and controlled release of anthocyanins from pharmaceutical formulations is important for gastrointestinal absorption, transepithelial transport, and intracellular uptake [16]. The release of anthocyanins from pharmaceutical dosage forms through a hydrophilic membrane was tested to assess their membrane permeability. The evaluation showed that anthocyanins passed through the hydrophilic membrane gradually, and that the total amount of anthocyanins released from the modeled gel formulations (G1–G9) during the first hour was within the range of 6.02%–13.50%. The gel formulation with 1% chitosan (G1) showed the fastest release rate and the highest amount of anthocyanins released (70% within 6 h). The other formulations showed a slower release of anthocyanins, and after 6 h, the amount of anthocyanins released from the formulations G2-G9 was <57%. The results of this study confirmed that the release of the active compounds from the semi-solid formulations depended on the base chosen [42]. Anthocyanins are unstable compounds, and slow and inadequate transport of these compounds results in their degradation due to pH changes and metabolism [43]. This explains why, under physiological conditions, only 1% of the individual anthocyanins consumed are detectable in plasma due to their inadequate absorption in the digestive tract [14].

A slower release of anthocyanins was achieved with the carbomer and sodium carboxymethyl cellulose gel formulations (G7 and G8) compared to the chitosan (G1–G3) and sodium carboxymethyl cellulose (G4–G6) gel formulations. Prolonged release of anthocyanins from gel formulations is promising for increasing their stability [16]. This is important, as undegraded anthocyanins reach the large intestine, where they are able to modulate the composition of the intestinal microbiota [12]. The colonic microbiota hydrolyzes anthocyanin glycosides into aglycones and breaks them down to phenolic acids, which lowers the intestinal pH and provides a carbon source for probiotic growth, thus creating a favorable environment for probiotics [44]. Anthocyanins also stimulate the production of bacteriocins and other substances that inhibit the production of harmful bacteria, reducing competition between the harmful bacteria and probiotics [44]. Anhê et al. studied the gut microbiota of mice fed cranberry extract at a dose of 200 mg/kg for 8 weeks and found an increase in the strain of *Akkermansia* spp. [45]. Liu et al. determined that cranberry anthocyanin extracts improved the growth of strains of the *Roseburia*, *Lachnoclostridium*, and *Clostridium innocuum* groups, and reduced the growth rates of *Rikenellaceae* and *Rikenella* bacteria in experimental mice [46]. Morato et al. determined, in a randomized controlled trial of healthy adults, that consuming 30 g of freeze-dried cranberry powder in the diet increased the population of the *Bacteroidetes* strains and decreased *Firmicutes* populations after five days of testing [47]. Alison Lacombe et al. found that cranberry anthocyanins were able to inhibit the growth of *E. coli* O157:H7 [48]. Anthocyanins have an important role in improving the composition of the microbiota by reducing inflammatory processes in the gut and the risk of diabetes, obesity, and cardiovascular disease [49,50]. The oral gel formulation allows for the creation of a readily consumable product that does not require additional preparation, while at the same time, such formulations can offer a controlled release of the active compounds, providing a useful alternative form of oral pharmaceuticals.

## 4. Conclusions

Cranberry fruit anthocyanins are released from semi-solid gelled matrices and permeate the hydrophilic membrane at different rates. Under physiological conditions, anthocyanins are degraded due to pH changes and metabolism, and prolonged release of active compounds can help solve the problems of anthocyanin instability and low bioavailability.

In order to model future controlled-release gel structures, it is important to pay attention to the selection of gelling agents and to assess the rheological properties of the gels. The dissolution kinetics of anthocyanins from the model gels depended on the ability of the selected carrier to dissolve in the acceptor medium. The in vitro dissolution test showed that the release of anthocyanins from the gel formulations with chitosan (G1–G3) and sodium carboxymethyl cellulose (G4–G6) was most intense within 10 min, and then varied slightly. This study showed that gel formulations with sodium carboxymethyl cellulose and carbomer (G7 and G8) showed a statistically significant longer dissolution rate and time to release through the hydrophilic membrane into the acceptor medium. These gel formulations provide a modified release of the active compounds and are therefore promising for the development of a modified-action oral gel that could stabilize anthocyanins and deliver them to the large intestine to modulate the composition of microbiota.

## 5. Materials and Methods

### 5.1. Reagents

All standards, reagents, and solvents used were of analytical grade. Deionized water was produced using a Milli-Q water purification system (Milli-Q^®^, Millipore, MA, USA). The standards used in the UPLC analysis (peonidin-3-galactoside, peonidin-3-arabinoside, cyaniding-3-galactoside, cyaniding-3-arabinoside, peonidin-3-glucoside, cyaniding-3-glucoside, cyanidin chloride, peonidin chloride, malvidin-3-galactoside, malvidin-3-arabinoside, and delphinidin-3-galactoside) were obtained from Extrasynthese (Genay, France). The chemicals applied in the modeling of gel formulations were sodium carboxymethyl cellulose, chitosan (deacetylation grade ≥ 75%, medium molecular weight), PEG400 sodium hydroxide (NaOH), and acetic acid, all obtained from Sigma-Aldrich Chemie GmbH (Steinheim, Germany). Carbomer 980 was purchased from Lubrizol (Wickliffe, OH, USA). Acetonitrile was purchased from Sigma-Aldrich (Steinheim, Germany), and formic acid was purchased from Merck (Darmstadt, Germany).

### 5.2. Preparation of Freeze-Dried Cranberry Extract

*Vaccinium macrocarpon* Aiton fruits of the cultivar ‘Stevens’ were harvested on 1 September 2022, in Lithuania (54°57′50.8″ N 23°01′34.7″ E). Cranberries were frozen at −60 °C in an ultra-low temperature freezer (CVF330/86, ClimasLab SL, Barcelona, Spain) and subsequently freeze-dried according to the methodology described by Urbstaite et al. The freeze-dried cranberry fruits were ground into a powder (particle size about 100 µm) with an electric grinder (Retsch GM 200, Retsch GmbH, Hahn, Germany). The freeze-dried cranberry fruit powder was extracted with hot water at a ratio of 1:50 in a percolator. The collected aqueous cranberry fruit extract was frozen at −60 °C in an ultra-low temperature freezer (CVF330/86, ClimasLab SL, Barcelona, Spain) and freeze-dried in a lyophilizer (Zirbus, Zirbus Technology GmbH, Bad Grund, Germany) at the condenser temperature of −85 °C and the pressure of 0.01 mbar. The cranberry fruit extract lyophilizate was ground into a powder (particle size about 100 µm) with an electric grinder (Retsch GM 200, Retsch GmbH, Hahn, Germany). Loss on drying was determined using the European Pharmacopoeia method [51].

### 5.3. UPLC-PDA Analysis for the Characterization of Anthocyanins

The analysis was conducted using the methodology that was developed and validated by Vilkickytė et al. (all system parameters corresponded to those specified in the article) [6]. The ultra-high-performance liquid chromatography (UPLC-DAD) analysis was carried out under the following procedures: 1 μL of anthocyanin solution was injected into the UPLC system and was separated under an ACE C18 reversed phase column, 100 × 2.1 mm, 1.7 µm particles, and was stored in a thermostat at 30 °C; elution was carried out with a binary high pressure gradient at a flow rate of 0.5 mL/min (solvent A: 100% acetonitrile (*v/v*); solvent B: aqueous 10% formic acid solution (*v/v*)). The mobile phases were eluted as follows: 95% A from 0 min to 2 min, 91% A from 2 min to 7 min, 88% A from 7 min to 9 min, 75% A from 9 min to 10 min, 20% A from 10 min to 10.5 min, 20% A from 10.5 min to 11 min, and 95% A from 11 min to 12 min. Anthocyanins were determined at 520 nm using Empower 3 software (Waters, Milford, MA, USA). The anthocyanin concentrations were measured using the calibration equation created from the standards.

### 5.4. Preparation of the Gel Formulations and Their Biopharmaceutical Evaluation

#### 5.4.1. Preparation of the Gels

The descriptions of the formulations of the gels are given in Table 3. Chitosan gel formulations were made by dissolving chitosan in water using a hotplate and adding the required amount of 30% acetic acid until the chitosan was completely dissolved to form a gel consistency. Dry cranberry extract was dissolved in the prepared chitosan base. The required amount of water was added to bring the gel to the desired consistency. Sodium carboxymethyl cellulose gels were prepared through swelling sodium carboxymethyl cellulose in a small amount of water. The sodium carboxymethyl cellulose was then dissolved in the required amount of water until the gel consistency was obtained. PEG400 was added to give softness to the gel, and dry cranberry extract was then dissolved. The preparation of the gel formulations with carbomer and sodium carboxymethyl cellulose was carried out by mixing the prepared carbomer gel and the prepared sodium carboxymethyl cellulose gel, followed by the addition of PEG for softness and the dissolution of the dry cranberry extract. All prepared experimental gels were kept in a refrigerator at 5 °C.

#### 5.4.2. Evaluation of the Physiochemical Properties of the Gels

The pH values of the gels were established with a pH meter (766 with a Knick SE 104N electrode, Berlin, Germany).

#### 5.4.3. In Vitro Dissolution Test

An in vitro dissolution test of the 5 g gel formulation was carried out using a dissolution tester (Sotax AT 7 smart, SOTAX AG, Allschwil, Switzerland). The samples were taken after 10 min, 15 min, 20 min, 30 min, 45 min, and 60 min. The test was conducted at a rotational speed of 100 rpm and at the temperature of 37 ± 0.5 °C. The acceptor medium was 250 mL of water. During this study, 0.3 mL of the sample was withdrawn from the dissolution media and was replaced with 0.3 mL of a fresh medium solution. The samples of the dissolution medium were filtered through a 0.2 µm pore size filter (PVDF Acrodisc LC 13, Pall Corporation, New York, NY, USA) into a chromatographic bottle insert. The samples were analyzed by applying the UPLC-PDA method described in Section 5.3.

#### 5.4.4. In Vitro Studies of Release from Gel

The estimation of the release of anthocyanins from edible gels was carried out using natural cellulose dialysis membranes (Franz type diffusion cells) (Sigma-Aldrich, St. Louis, MI, USA). The donor compartment contained 1 g of the gel formulations (G1–G9). The acceptor compartment contained water at 37.0 ± 0.5 °C. A dialysis tubing cellulose membrane was inserted between the acceptor and the donor chambers. The acceptor solution was continuously mixed with the hotplate magnetic stirrer (IKAMAG C-MAG HS7, IKA-Werke GmbH & Co. KG, Staufen, Germany). During this study, 0.2 mL of the sample was withdrawn from the acceptor media and was replaced by 0.2 mL of a fresh medium solution. The samples were taken after 1 h, 2 h, 3 h, 4 h, 5 h, and 6 h. Samples of the dissolution medium were filtered through a 0.2 µm pore size filter (PVDF Acrodisc LC 13, USA) into a chromatographic bottle insert. The samples were analyzed by applying the UPLC-PDA method described in Section 5.3.

### 5.5. Data Analysis

Statistical analysis was performed using the Kruskal–Wallis 1-way ANOVA (k samples) test with a multiple comparisons (stepwise step-down) test employing the IBM SPSS Statistics 29.0 software (IBM, Armonk, NY, USA). Statistical analysis was used to assess the difference in anthocyanin levels between test samples, which were considered significantly different if *p* < 0.05. The data were presented as means ± standard deviations, and in vitro simulations were conducted in triplicate. The graphs were produced with Microsoft Office Excel 2021 (Microsoft, Redmond, WA, USA).

## Figures and Tables

**Table 1:** Content of anthocyanins and anthocyanidins in large cranberry extract.

Compound	Amount, µg/g
Delphinidin-3-galactoside	10.75 ± 0.2
Cyanidin-3-galactoside	1469.46 ± 22.0
Cyanidin-3-glucoside	32.79 ± 0.5
Cyanidin-3-arabinoside	947.66 ± 14.2
Peonidin-3-galactoside	1976.20 ± 29.6
Peonidin-3-glucoside	105.35 ± 1.6
Peonidin-3-arabinoside	668.11 ± 10.0
Malvidin-3-galactoside	9.73 ± 0.2
Malvidin-3-arabinosie	7.73 ± 0.1
Peonidin	5.17 ± 0.1
Cyanidin	8.37 ± 0.1
Sum of compounds	5241.32 ± 78.6

**Table 2:** Organoleptic characteristics and pH values of the gels.

Formulation no.	G1	G2	G3	G4	G5	G6	G7	G8	G9
Organoleptic characteristics	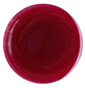	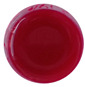	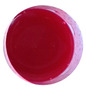	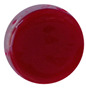	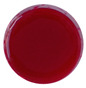	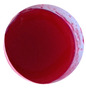	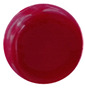	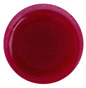	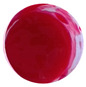
Red homogeneous gel with a weak cranberry odor									
pH value	2.95 ± 0.31	2.80 ± 0.31	2.91 ± 0.31	2.93 ± 0.31	2.95 ± 0.31	2.86 ± 0.31	3.10 ± 0.31	3.36 ± 0.31	3.09 ± 0.31

**Table 3:** Compositions of edible gels with cranberry extract.

Formulation no.	Cranberry Extract, g	Chitosan, g	NaCMC, g	Carbomer, g	PEG400, g	NaOH, 10% (*w*/*v*)	Acetic Acid, 30% (*v*/*v*)	Water, g	Content, g
G1	15.0	1.0	–	–	–	–	10–15 drops	ad 100	100.0 ± 0.5
G2	15.0	2.0	–	–	–	–	10–15 drops	ad 100	100.0 ± 0.5
G3	15.0	3.0	–	–	–	–	10–15 drops	ad 100	100.0 ± 0.5
G4	15.0	–	2.0	–	10.0	–	–	ad 100	100.0 ± 0.5
G5	15.0	–	3.0	–	10.0	–	–	ad 100	100.0 ± 0.5
G6	15.0	–	4.0	–	10.0	–	–	ad 100	100.0 ± 0.5
G7	15.0	–	2.0	1.0	10.0	2–3 drops	–	ad 100	100.0 ± 0.5
G8	15.0	–	3.0	1.0	10.0	2–3 drops	–	ad 100	100.0 ± 0.5
G9	15.0	–	4.0	1.0	10.0	2–3 drops	–	ad 100	100.0 ± 0.5

## Data Availability

All data generated during this study are included in this article.
